# Therapeutic and Supportive Effects of Cannabinoids in Patients with Brain Tumors (CBD Oil and Cannabis)

**DOI:** 10.1007/s11864-022-01047-y

**Published:** 2023-01-12

**Authors:** J. Eduardo Rodriguez-Almaraz, Nicholas Butowski

**Affiliations:** 1 Neuro Surgery Department Division of Neuro-Oncology, University of California San Francisco, 400 Parnassus Avenue, 8th floor, RM A808, San Francisco, California USA; 2 Deparment of Epidemiology and Biostatistics, University of California San Francisco, 400 Parnassus Avenue, 8th floor, RM A808, San Francisco, California USA; 3 Deparment of Molecular Science, University of California San Francisco, 400 Parnassus Avenue, 8th floor, RM A808, San Francisco, California USA

**Keywords:** Cannabinoids, Gliomas, Palliative care, Antitumor therapy, Symptom management

## Abstract

The potential medicinal properties of *Cannabis* continue to garner attention, especially in the brain tumor domain. This attention is centered on quality of life and symptom management; however, it is amplified by a significant lack of therapeutic choices for this specific patient population. While the literature on this matter is young, published and anecdotal evidence imply that cannabis could be useful in treating chemotherapy-induced nausea and vomiting, stimulating appetite, reducing pain, and managing seizures. It may also decrease inflammation and cancer cell proliferation and survival, resulting in a benefit in overall patient survival. Current literature poses the challenge that it does not provide standardized guidance on dosing for the above potential indications and cannabis use is dominated by recreational purposes. Furthermore, integrated and longitudinal studies are needed but these are a challenge due to arcane laws surrounding the legality of such substances. The increasing need for evidence-based arguments about potential harms and benefits of cannabis, not only in cancer patients but for other medical use and recreational purposes, is desperately needed.

## Introduction

Brain tumors mostly arise from glial cells (as opposed to neuronal), like astrocytes, and are graded on a World Health Organization scale from 1 to 4, where each increase in number reflects a higher grade of malignancy [1]. Astrocytoma is the most frequent glial primary brain tumor among more than 150 different types and it comprises about 60% of all primary brain tumor diagnoses [2]. High-grade or malignant central nervous system (CNS) tumors are among the most fatal cancers, and they have not had successful therapies developed over the past two decades.

The overall incidence of malignant brain tumors varies across different regions of the globe. In 2016, it was estimated that there were 330,000 new cases (4.63 per 10,000 person-rate [4.17 to 4.90]) with 227,000 deaths (95% CI: 205,000 to 241,000) attributed to CNS cancer globally. The geographical regions with higher incidence per 100,000 person-years include east Asia (98,000 to 122,000), western Europe (37,000 to 54,000), and south Asia (29,000 to 37,000) [3]. In 2021, it was estimated that more than 84,000 people were diagnosed with a primary brain tumor (including gliomas) in the United States of America (USA) [4].

The survival rate at 5 years for all malignant gliomas combined has increased due to development of new therapies: 36% in the period observed between 2009 and 2015 compared to 23% from 1975 to 1977. However, the specific 5-year survival rates vary across populations and glioma subtypes. Glioblastoma (aka astrocytoma grade 4), however, remains one of the most aggressive forms of cancer, with a median survival after resection, radiotherapy, and chemotherapy of 12 to 15 months and a 5-year relative survival rate of ~ 5% [5].

In general, treatment of brain tumors initially consists of maximally safe surgical resection followed by radiation therapy and concurrent chemotherapy (often temozolomide), followed by adjuvant chemotherapy that varies depending on the tumor subtype, location, size, patient, and oncologist’s preference. At recurrence, the course of treatment depends on a number of factors that include the molecular features of the tumor as well as the patient’s clinical status and personal preferences toward repeat surgery, more radiation, chemotherapies, or clinical trials [5, 6].

All of the above factors (high heterogeneity, treatment complexity, and treatment limitations) contribute to the challenge of symptom management whose etiology can be inherent to the tumor itself or result from the side effects of the therapies employed. Additionally, symptom severity varies widely, depending on the size and location of the tumor, as well as the past medical history of any given patient. Among the most frequent symptoms, patients may experience headaches, vision impairment, hearing impairment, speech difficulty, and balance difficulty [7]. As treatment ensues, or the cancer grows, other complications such as fatigue, depression, memory, or personality changes decline in mental function or brain capacity, seizures, and muscle paralysis might be present.

In this review, we provide the reader with a broad view of the current landscape of brain tumor treatment, the role of cannabis in the brain tumor context, and how the patients’ quality of life and therapeutic efficacy may be improved by the use of cannabis.

## Cannabinoid signaling

Cannabinoids are a group of structurally heterogeneous and complex compounds [8•, 9]. Here we will consider cannabinoids within their three main subtypes: phytocannabinoids (plant derived), synthetic cannabinoids, and endocannabinoids.

The *Cannabis* plant produces more than 700 different chemical compounds, including 120 phytocannabinoids, but fewer than 50 of these compounds are produced in significant amounts, while the rest are found in negligible amounts [8•]. Cannabinoids contained in herbal cannabis preparations or whole-plant extractions tend to work synergistically to elicit the various effects associated with its consumption. As such, it would be erroneous to adjudicate the most medicinal value to a single phytocannabinoid. However, delta-9-tetrahydrocannabinol (THC) has been the most studied and is recognized as the most potent of the phytocannabinoids [9]. Additionally, cannabidiol (CBD) has been identified as an important compound, which has led to an increased focus of research into this compound. As part of the effort to understand the biological effects of phytocannabinoids in humans, recent studies have shown that they possess anticancer properties through inhibition of cell proliferation, migration, and angiogenesis, as well as induction of apoptosis of cancerous cells [10•]. The wide range of these observed medicinal effects has been attributed to the plant variant heterogeneity that possess distinct ratios of active compounds [8•].

In contrast, synthetic cannabinoids (SCBs) are manmade chemicals with high affinity to cannabinoid receptors (CB_1_ and CB_2_) and are considered direct agonists of these receptors, whereas THC is a partial agonist. Due to this property, SCBs have a highly toxic (often unpredictable) profile and have a high potential for abuse. For these reasons, authorities (federal, state, and local) have made it illegal to sell, buy, or possess the primary chemical components used to manufacture SCBs, and, in turn SCBs themselves [11].

Given the lack of medical benefit, it is important to distinguish between SCBs and other cannabinoids, and SCBs will not be the focus of the present publication.

Endocannabinoids are pleiotropic signaling molecules involved in a variety of processes that include re-establishing homeostasis after pathological insults. Since the identification of the endogenous cannabinoid receptors CB_1_ and CB_2_, they have been described as the primary components of the endocannabinoid system along with their ligands, such as peroxisome proliferator receptors (PPARs), adenosine receptors, and G-coupled proteins (GPR55). CB_1_ is a G protein-coupled receptor (GPCR) that is abundantly expressed in the brain. In contrast, CB_2_ (also GPCR) is found to be highly expressed in the immune system. Additionally, two arachidonic acid derivatives, 2-arachidonoylglycerol (2-AG), and arachidonoylethanolamide (AEA) have been identified in the brain and intestinal tissues, and they have shown high affinity and efficacy to activate CB_1_ and CB_2_. Although some other amides, esters, and long-chain polyunsaturated fatty acids also exhibit cannabimimetic properties [12], 2-AG and AEA have been characterized as the main endocannabinoids [12, 13].

Additionally, genetic manipulation of cannabinoid receptors in mouse models suggested increased susceptibility to neurodegenerative disorders, which was later confirmed in human subjects [14, 15] suggesting the targeting of components of the endocannabinoid system as a possible therapeutic strategy for cancer and associated symptoms [18].

### Cannabinoid system in brain tumors

A growing body of evidence suggests that alterations to the balance between the levels of endogenous cannabinoid ligands and their receptors occur during malignant transformation in various types of cancer, including brain tumors [16]. As do most immune cells in the body, non-cancerous astrocytes express only the CB_1_ receptor; however, both functional cannabinoid receptors have been identified in several human glioblastoma cell lines as well as in primary cultures derived from glioblastoma [17]. Additionally, expression of the CB_2_ receptor was shown to be increased in tumoral cells and invading macrophages and tumoral endothelial vessel cells [18, 21]. As such, a positive correlation between the tumor malignancy grade and levels of CB_2_ receptor expression has been observed, showing the highest level of CB_2_ expression among IDH-wild-type Glioblastoma grade 4 (as per WHO 2021 classification) [10•]. These observations imply that high expression levels of CB_2_ receptor in brain tumors make them particularly susceptible to the antitumor effects of circulating endocannabinoids like AEA, resulting in tumor involution [19]. In support of this hypothesis, inhibitors of endocannabinoid transport and degradation have been shown to suppress tumor progression in some types of cancer, including brain tumors [20]. Thus, the identification of differential expression of cannabinoid receptors in glioblastomas has driven the exploration of therapeutic strategies, including the increasing the expression of CB_1_ and CB_2_ receptors, increasing the bioavailability of endocannabinoids (via the minimization of synaptic degradation), as well as increasing biosynthesis and uptake [21, 22].

### Potential symptom-based applications of cannabis in patients with brain tumors

#### Chemotherapy-induced nausea and vomiting (CINV)

Nausea and vomiting can present in an acute, subacute, delayed, or anticipatory fashion in patients undergoing chemotherapy. It is one of the most common toxicities and is, by far, the most common disease symptom considered by patients as the most stressful and cumbersome side effect [23•]. Early results of our own observational experience show that the relative unpredictability of the severity of CINV has the potential to cause depression, anxiety, and mood changes given the potential disruption to the daily life of patients undergoing chemotherapy treatment [24]. Cannabinoids are well known to exert antiemetic effects in cancer patients [25]. As such, the anti-CINV effect of cannabis is one of the better established properties [26, 28]. Given this well-characterized effect and the fact that it is widely known, observational studies like ours have shown that nausea and vomiting are one of the main reasons for which cancer patients start cannabinoid use (including medical marijuana) [23, 27]. A growing body of evidence [28] has shown that cannabinoids—THC in particular—are more effective than traditional antiemetics (prochlorperazine, metoclopramide, chlorpromazine, haloperidol), including serotonin (5-HT_3_) receptor antagonists (such as ondansetron) to treat moderate nausea produced by chemotherapy, but not as effective for severe emetogenic treatments [28]. As a result, dronabinol was first approved by the FDA in 1986 to be used as treatment of CINV. In contrast, nabilone and levonantradol did not show superior acute antiemetic efficacy when compared to standard antiemetic therapies [29]. This has led to low dose cannabinoids to be included as recommended compounds (with respective caution) to treat emesis in the latest National Comprehensive Cancer Network Guidelines published in 2022 [30] with the added benefit that cannabinoids act as appetite stimulants.

#### Cannabinoids as appetite stimulant for tumor cachexia control

Tumor cachexia and weight loss are a well-known problem in oncology in general, and it is a common concern among brain tumor subjects. It is estimated that approximately 50–80% of cancer patients suffer from cancer cachexia syndrome and it has been implicated in 25% of cancer deaths [31]. Additional, malnutrition due to the side effects of chemotherapy and radiotherapy may further lead to a number of metabolic imbalances that negatively impact the overall clinical outcomes and quality of life of patients. In recent years, there has been gradual but steady progress in unveiling the benefits of cannabinoid usage as a supportive therapy and in palliative medicine [32]. Although the majority of trials exploring the orexigenic effect of cannabinoids has been observed in HIV patients [33], in 2011, Brisbois and collaborators demonstrated that THC improves chemosensory perception, alters macronutrient preference, and increases appeal of savory foods and appetite in patients with advanced cancer [34]. Currently, dronabinol is the only cannabinoid that has been approved by the FDA to treat weight loss associated with anorexia in patients diagnosed with AIDS and to treat nausea and vomiting associated with cancer chemotherapy in patients who have failed to respond adequately to conventional antiemetic treatments [35]. Altogether, this evidence suggests that cannabinoids have a great potential to improve nutritional intake, the overall health status, and overall quality of life of brain tumor patients.

#### Seizure control

Seizures are a major component of the spectrum of symptoms of patients with brain tumors, and they are often the first symptom that patients with no history of seizures report at initial diagnosis [36]. Depending on the location, size, and shape of the tumor, seizures are often difficult to treat leading to significant detriment in patients’ health outcomes and overall quality of life [7]. Additionally, seizures may have an additive effect to the neurocognitive deterioration that some brain tumor patients (depending on location, grade, and size of tumor) experience. Counterintuitively, the sole management of seizures using standard treatments is not enough to slow or prevent neurocognitive decline given the inherent side effects of the drugs employed (somnolence, dizziness, agitation, anxiety, irritability, depression, headache, attention disturbance, etc.) [37]. Management of brain tumors with surgery, radiotherapy, and chemotherapy may contribute to seizure control (via tumor treatment), but tumor-related epilepsy is often refractory despite adequate treatment with standard anti-epileptic medications [38]. Research efforts to explore the effects of cannabinoids in seizure and epileptic disorders led to the approval of cannabidiol (Epidiolex) in oral solution for the treatment of seizures associated with two rare and severe forms of epilepsy in children [39].

In the neuro-oncology field, only anecdotal data has been observed to date regarding the anti-epileptic properties of cannabis [40]. However, growing research efforts in this area suggest that cannabinoids may play an important role in seizure control with few side effects [41]. Another important consideration that is carefully weighted when prescribing anti-seizure medications by neuro-oncologists is their significant effect on the cytochrome P450 (CYP) enzyme system, which may have effects on the metabolism of numerous chemotherapeutic agents interfering with tumor treatment itself. As such, when possible, enzyme-inducing anti-epileptic drugs (IADs) are avoided and non-EIADs are preferred [6].

While cannabinoids have a great potential as anticonvulsants in brain tumor patients, both CBD and THC are potent inhibitors of CYPs and can profoundly affect drug bioavailability and disposition [42]. Thus, it is important to evaluate the risk-benefit ratio when using cannabinoids in brain tumor patients undergoing chemotherapy alone or chemoradiation.

#### Cannabinoid use for pain management

Another symptom related to cancer in general (not limited to brain tumors) is chronic pain [36]. Additionally, chemotherapeutic agents can induce neurotoxic side effects that include severe neuropathic pain [43], which reduces patient function and overall quality of life. Neuropathic pain associated with chemotherapy (e.g., cisplatin, carboplatin, vincristine, and paclitaxel) is particularly resistant to treatment and it remains a problem as chemotherapeutic regimens become more successful in extending peoples’ life. Agents such as vincristine sensitize neurons in the spinal cord that carry information about pain, providing a neural basis for chemotherapy-evoked neuropathic pain. Preclinical studies have shown that cannabinoid agonists suppress established neuropathic pain (central and peripheral) induced by chemotherapy treatment [44–47], which establishes the basis for possible underlying mechanisms that support the clinical use of cannabinoids in management of chemotherapy-induced pain. Further preclinical evidence has shown cannabinoids as promising suppressants of neuropathic pain evoked by all major classes of chemotherapeutic agents, including platinum and vinca alkaloid agents [48], all common agents used to treat brain tumors. In fact, multiple mouse and cell models have shown that CB1 and CB2 receptor agonists are effective at reducing chemotherapy-induced neuropathic pain by modulating a variety of mechanisms including inhibition of calcium channel activity, transient receptor potential channels, serotonin, GABA and glutamate receptor signaling, and modulation of the immune system, leading to anti-inflammatory activity [49]. Moreover, a series of observations made by D’Andre et.al. have suggested that topical cannabinoids may be helpful for patients with chemotherapy-induced peripheral neuropathy [50]. A review conducted in 2015 that included 3 cannabinoids (THC, nabiximols, and nabilone) found moderate-quality evidence to support the use of cannabinoids for the treatment of chronic pain and spasticity [51]. Researchers reported that the average number of patients who reported a reduction in pain of at least 30% was greater in the cannabinoid group compared with the placebo group. Additionally, it was observed that the analgesic effects of THC were dose dependent (with tested doses up to 60 mg of THC). In an open-label clinical trial on patients with cancer-related pain who experienced inadequate analgesia despite the use of opioids, patients were assigned to three treatment groups: THC/CBD spray, THC spray, or placebo. Researchers found that patients reported a constant improvement in the TH/CBD spray group, they also observed a sustained effect despite long-term use, which allowed them to keep their standard analgesic medications without increasing their doses [52]. Although further research is urgently needed in the context of brain tumors, the body of evidence presented suggests that cannabinoids are compounds with a safer profile than opioids that may be used in pain management among brain tumor patients.

#### Neuroprotective effect

As mentioned above, therapeutic decisions for brain tumor patients include extent of surgical resection, radiation dose fields, and chemotherapy regimen. Neuro-oncologists often make the decision of a specific therapeutic plan in conjunction with the patient, weighing the impact that the therapy will have on the cognitive and overall neurologic function of the patient. As such, therapies that provide neuroprotection are highly valued in the brain tumor context.

Chemotherapy-induced cognitive impairments can be observed not only during pharmacological treatment of the disease, but also long after treatment cessation. The biological mechanisms that are implicated in the cognitive decline induced by chemotherapy include direct neurotoxic effects, impaired neurogenesis, increased neuronal degeneration, white matter abnormalities, inflammatory reactions, and increased oxidative stress activity [53]. Similarly, preclinical evidence in a wide variety of animal models has shown that chemotherapeutic agents such as paclitaxel (among others) can induce short- and long-term deleterious effects in executive and non-executive functions (such as working memory and spatial learning) [54, 55]. Furthermore, clinical neuroimaging studies have shown alterations of brain structure and plasticity in patients who received chemotherapy. These studies have shown the presence of cognitive alterations regardless of tumor location, suggesting that chemotherapy treatment may induce alterations that contribute to the cognitive decline of patients [56].

While the investigation of the possible positive impact of the employment of cannabinoids as neuroprotective agents has gained attention in recent years, the progress made is only in the preclinical setting. This is, in part, because neurocognitive deficiencies are difficult to detect using standard assessments since the neurological function level observed in patients undergoing chemotherapy often falls within the lower quartiles compared to the neurotypical population. Additionally, the lack of standardized tests to detect these deficiencies complicates not only the medical evaluation but also the cognitive rehabilitation [57].

Due to the ubiquitous distribution of cannabinoid receptors and the complexity of the signaling of the endocannabinoid system, cannabinoids have been proposed as modulators of cognitive functions indicating, for example, that cannabinoids could be employed as anxiolytics at low doses in animal models [15]. While the clinical evidence in this space is lacking, THC and nabilone have been formally proposed as co-adjuvants in the treatment of depression and anxiety [26]. Furthermore, the endocannabinoid system has been implicated in the modulation of the immune system which can promote neurogenesis and slow neuro-degeneration [58]. Cannabinoids have been used as therapeutic tools in pathologies that combine neuroinflammatory responses and cognitive impairments such as Parkinson’s disease [59], Alzheimer’s disease [60], or traumatic brain injury [61]. Specifically, agonists of CB_1_ and CB_1_/CB_2_ agonists (such as HU-210 and WIN_55,12-2_) exhibited a reduction of microglial activation and, in turn, pro-inflammatory cytokine expression [15]. Additionally, an increase in CB_2_ receptor expression has been positively correlated with an increase of microglial activation in animal models of neuroinflammation and neurotoxicity, which was correlated to neuroprotection [62], shedding light on the neuroprotective effects of cannabinoids. Despite this body of evidence, the clinical experience in glioma and chemotherapy-induced cognitive impairment is lacking and trials exploring this are needed. Although observational, our experience had led us to identify that patients who use cannabinoids show a slower cognitive decline over time compared to non-users. Additionally, cannabis users improved their mood and social functioning as measured by standardized scales [40].

## Antitumoral effects of cannabinoids in brain tumors

### Cell growth and proliferation control

Cannabinoids have the potential to induce significant inhibition of cell growth in tumor cells via modulation of proteins and nuclear factors involved in the control of cell survival, transformation, and cell death [63]. An example of this signaling cascade is the inhibition of adenylyl cyclase via CB_1_ and CB_2_ receptor activation and subsequent G_i/o_ activation. This, in turn, triggers different metabolic pathways such as mitogen-activated protein kinase (MAPK), phosphoinositide 3-kinase (PI3K), and cyclooxygenase-2 pathways (COX-2), as well as accumulation of ceramide, modulation of protein kinase B (Akt), and ion channels [64] preventing cellular growth. Such pathways are well-known cell regulation and proliferation pathways.

As such, apoptosis of glioma cells promoted by cannabinoid treatment was first described by Manuel Guzman in 1998 [65]. The apoptotic effects triggered by a synthetic agonist of CB_1_ and CB_2_ (WIN 55,212-2) were observed in cell cultures derived from rat glioma cells [67]. Specifically, local administration of THC or WIN55,212-2 reduced the size of tumors generated by intracranial inoculation of C6 glioma cell lines in rats, leading to the complete eradication of gliomas and increased survival in one-third of the treated rats [66]. Altogether, this evidence suggests that cannabinoids have a great potential to be used as efficacious adjuvants in the treatment of brain tumors.

### Pro-apoptosis

As mentioned before, the activation of CB_1_ and CB_2_ receptors trigger different signal pathways that participate in cannabinoid-induced cell death in various tumor cells including gliomas [26]. A possible mechanism that has been proposed is the sustained accumulation of sphingolipid ceramide (a pro-apoptotic lipid), which modulates signaling pathways that are crucial in the control of tumor cell growth and cell survival [67]. Additionally, increased ceramide levels induced by cannabinoids leads to prolonged activation of the Raf-1/MEK/ERK signaling cascade, which mediates the arrest of cell cycle in glioma cells inhibiting cell proliferation and promoting apoptosis [64, 67].

Moreover, phytocannabinoids have been shown to trigger apoptosis in glioma cells via oxidative stress, endoplasmic reticulum (ER)-stress, and autophagy [65]. Specifically, evidence has shown that THC activates ER-stress that ultimately results in apoptotic death by altering balance between ceramides and dihydroceramides. Furthermore, this effect also promotes autophagy and, in turn, it activates the caspase cascade triggering apoptosis. The activation of this pathway is essential for the antitumor effect of THC observed in glioma xenografts [17].

Interestingly, synergistic effects of THC and other cannabinoids (CBD and cannabigerol (CBG)) have shown to be able to inhibit the ability of cancerous cells to proliferate, become viable, and invasive in in vivo experiments [68]. In contrast, evidence suggests that sub-lethal doses of THC lead to cell proliferation dependent on the activity of metalloprotease, endothelial growth factor (EGFR), and tumor necrosis factor α-converting enzyme (TACE/ADAM17) that mediates EGFR transactivation. This apparent “reversal” of the expected effect of THC seems to be critically dependent on THC concentrations and EGFR transactivation [69]. Thus, the proliferative effect at low concentrations of THC suggests that patients may benefit from high continuous doses of THC, and this should be considered because of the interaction of THC (and other cannabinoids) with chemotherapy drugs [16].

### Anti-angiogenic effects of cannabis

It is well known that rapidly growing glioma cell populations have high metabolic demands. This increase in metabolic demand necessitates a new vasculature supply, making angiogenesis blockade one of the most important antitumoral strategies explored in recent years [70]. As brain tumors progress, the vascular demand grows due to hypoxic conditions triggering robust neoangiogenesis via the vascular endothelial growth factor (VEGF) and angiopoietin-2 pathways [71]. Cannabinoids have shown to impair the VEGF pathway by blunting VEGF production and signaling, as well as VEGF receptor 2 blockade (the most prominent VEGF receptor in glioma) [72].

While brain tumor therapies have improved the efficacy and safety profile over the last decade, these are not innocuous and have the potential of importantly impacting the quality of life of patients by inducing symptoms that include nausea, vomiting, liver damage, hematologic anomalies, pulmonary damage, cardiac diseases, asthenia, mucosal damage, and neurological damage [73]. Cannabinoids have shown a favorable safety profile in preclinical studies (in vivo and in vitro) [10], as well as from pilot clinical trials in patients with recurrent brain tumors. Additionally, cannabinoids have shown important potential not only for counteracting side effects from standard therapy (such as chemoradiation) [74, 75•], but they also have shown potential synergistic properties that position them as tumor treatment adjuvants, by promoting survival of healthy glial cells and neurons in different models of injury and suggesting additional potential neuroprotective effects of cannabinoids during radiation [26].

## Clinical studies of cannabinoids in brain tumors

One of the first clinical studies to directly assess the cannabinoid antitumoral effect in brain tumors was conducted by Guzman and collaborators in 2003. Researchers identified nine patients diagnosed with recurrent GBM and then administered an intratumoral THC solution. However, investigators were unable to observe clear differences in patient survival after 15 days of administration. But, they described decreased tumor cell proliferation and increased tumor cell apoptosis, which is consistent with observations made in preclinical models. Additionally, treated patients showed little to no psychotropic symptoms even though THC was delivered directly into the tumor cavity [76].

Although the risks and benefits of cannabinoid use in patients with established glioma have not been studied on a large scale, a placebo-controlled trial of nabiximols (*n* = 27) conducted by Twelves et al. showed that, in general, cannabinoids are well tolerated and that their safety profile is acceptable with few side effects [77•]. Additionally, this study showed a significant improvement of survival at year 1 for the nabiximols (an oromucosal spray) group (83%) compared to the placebo group (44%; *p* > 0.05) signaling that there is a benefit of cannabinoids in the treatment of glioma. These results were aggregated on a systematic review and meta-analysis exploring the literature that included clinical-level data in which we showed that, although limited, cannabis improves survival among treated patients [78].

More recently, Schloss and collaborators conducted a relatively large phase 2 randomized blinded clinical trial to assess different ratios of medicinal cannabis in patients with high-grade gliomas. The study included 88 patients with recurrent or inoperable high-grade glioma who were treated with a single dose per day of oil-based organic plant extracts of cannabis based on a 1:1 and 4:1 ratio of THC:CBD. The treatment was well tolerated and the main side effects included muscle spasms and hallucinations (3.4%) which are well-known side effects of cannabinoids. Similar to our observations, the authors found improvement in d quality of life, sleep quality, functional well-being, and contentment with quality of life scales among cannabis users [79•].

Altogether, this evidence suggests that medical cannabis and cannabinoids could become a therapy of choice in modern neuro-oncology; however, further studies are needed to establish not only safe but also efficacious doses.

## Conclusion

In recent years, the medicinal properties of the *Cannabis* genus have received increased attention around the world. This attention combined with the lack of therapeutic choices and side effect management from such therapies has greatly increased the interest of cannabis in brain tumor patients due to its safety profile.

Current evidence suggests medicinal cannabis may inhibit chemotherapy-induced nausea and vomiting, stimulate appetite, reduce pain, and decrease inflammation and cancer cell proliferation and survival [80]. In this context, cannabinoids have not only shown to possess properties that may help minimize the polypharmacy effect that chemotherapeutic agents entail but to be a potential co-adjuvant to these therapies.

Cannabinoid therapies including marijuana and CBD products may be a useful adjunct to steroids and palliative therapies to improve symptom management.

The increasing need for evidence-based arguments about potential harms and benefits of cannabis use paralleled a surge in empirical studies investigating the health impact of cannabis use, the majority of which are observational and focused upon negative health effects and addiction [81]. Although there have been some interventional studies to try to understand the impact of cannabinoids in gliomas the use by the public in general has greatly surpassed the scientific knowledge derived from clinical trials. As such, more rigorous studies aimed to understand the overall clinical impact of cannabis and cannabinoids in patients with brain tumors and the interactions of these compounds with standard of care treatments are urgently needed.
